# The Role of Vascular Endothelial Growth Factor in Small-airway Remodelling in a Rat Model of Chronic Obstructive Pulmonary Disease

**DOI:** 10.1038/srep41202

**Published:** 2017-01-24

**Authors:** Lu Wang, Zhibo Xu, Bin Chen, Wei He, Jingxian Hu, Liting Zhang, Xianzhong Liu, Fang Chen

**Affiliations:** 1 Zhejiang Chinese Medical University, Hangzhou, 310053, People’s Republic of China; 2 Department of respiration, The Second Affiliated Hospital of Zhejiang Chinese Medical University, Hangzhou, 310006, People’s Republic of China; 3 Respiratory physiology Laboratory, The First Affiliated Hospital of Zhejiang Chinese Medical University, 310006, People’s Republic of China; 4 Department of respiration, Shaoxing Hospital of Traditional Chinese Medicine, Shaoxing, 312000, People’s Republic of China; 5 Department of respiration, Dongyang Hospital of Traditional Chinese Medicine, Jinhua, 322100, People’s Republic of China

## Abstract

Small-airway remodelling is one of the most remarkable pathological features of chronic obstructive pulmonary disease (COPD), in which angiogenesis plays a critical role that contributes to disease progression. The endothelial cell-specific mitogen vascular endothelial growth factor (VEGF), as well as its receptors, VEGFR1, VEGFR2, are thought to be the major mediators of pathological angiogenesis, and sunitinib exhibits anti-angiogenesis property through VEGF blockage and has been widely used to treat various cancers. In our study, Sprague-Dawley rats were subjected to lipopolysaccharide (LPS) injection and cigarette smoke (CS) inhalation to induce COPD, following sunitinib administration was conducted. Haematoxylin-eosin, Masson staining and immunostaining analysis were used to evaluate the pathological changes; quantitative real-time PCR and enzyme-linked immunosorbent assay were performed to provide more compelling data on the function of VEGF, VEGFR1, VEGFR2 in angiogenesis. Sunitinib treatment was associated with less angiogenesis in small-airway remodelling with a slightly disordered lung architecture, and lower expression level of VEGF, VEGFR1, VEGFR2. Overall, our results indicate that VEGF is a vital important factor that contributes to the small-airway remodelling in a rat model of COPD through promoting angiogenesis, which mainly depend on the specific binding between VEGF and VEGFR1 and can be effectively attenuated by sunitinib.

Chronic obstructive pulmonary disease (COPD) is defined by the Global Initiative for Chronic Obstructive Lung Disease as a disease state characterised by persistent and progressive airflow limitation accompanied by increasing chronic inflammation in the airways and lungs due to noxious particles or gases^1^, which remains a serious public health problem worldwide because of its high morbidity and mortality^2^. Participants who entered one study without COPD at the age of 40 years had a 12.7% (men) or 8.3% (women) risk of developing COPD within the next 40 years. After 1 year of follow-up, 26.0% of patients with severe COPD and 2.8% of those without COPD died^3^. COPD causes serious economic and social burdens and is estimated to become the third leading cause of death worldwide by 2020^4^. Multiple factors have been found to contribute to the pathogenesis and prognosis of COPD, and new therapeutic strategies and medications are still required for treatment of this disease. Angiogenesis, mediated by multiple growth factors with complementary and coordinated functions, plays a pivotal role in small-airway remodelling in patients with COPD^5^. Vascular endothelial growth factor (VEGF) and its receptors (VEGFR1/Flt-1 and VEGFR2/KDR/Flk-1) are considered to be the key regulators of both physiological and pathological angiogenesis^6^. In the present study, we explored the role of VEGF, VEGFR1, VEGFR2 in the small-airway remodelling associated with lipopolysaccharide (LPS) injection and cigarette smoke (CS) inhalation induced COPD and prioritized investigation of the curative effect of sunitinib, a typical tyrosine kinase inhibitor, in the treatment of COPD.

## Results

### The General condition of rats were observed

During the experimental period, the fur of the COPD rats turned yellow and exhibited less lustre, tended to more sensitive to the surrounding environment at week 10. When it was time to week 15, the rats exhibited nostril flaring and occasional diarrhoea. At week 20, the rats became nervous and manic, and some exhibited an obvious decrease in eating, drinking and weight. Rapid breathing and diarrhoea were seen frequently. In contrast, the normal rats stayed strong and fit, without any anxiousness or diarrhoea. No rat died during the study.

### Small-airway remodelling with serious disordered lung architecture is a prominent feature in COPD rats induced by LPS injection and chronic CS inhalation

**Figure 1:**
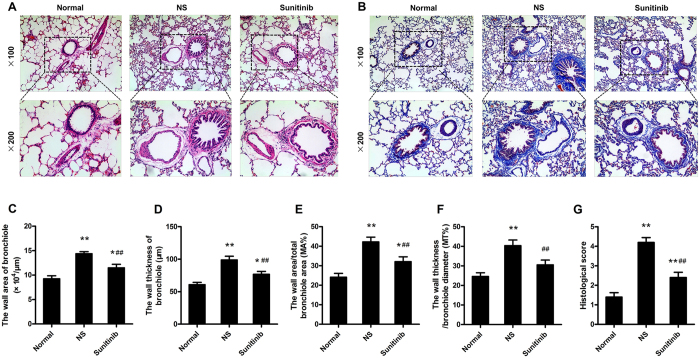
Small-airway remodelling in a rat model of COPD. (**A**) Representative photomicrographs of lung sections obtained from normal rats and COPD rats (HE; original magnification ×100, upper panels; ×200, lower panels). LPS injection and chronic CS inhalation resulted in septal congestion and increased numbers of inflammatory cells accumulation, more neutrophils, macrophages, and lymphocytes compared to the normal rats. It could be also noted the serious thickened tracheal epithelium and bronchiole walls with the presence of damaged alveolar walls and pulmonary bullae. Remodelled bronchiole and narrow tube caliber surrounded by proliferative connective tissue lead to the massive disruption of lung structure and irreversible airflow limitation that similar to the clinical patients of COPD. (**B**) A selected representative images of lung sections in each group (Masson staining; original magnification ×100, upper panels; ×200, lower panels) reveals the increased extracellular matrix (ECM) proteins deposition and fibrosis changes. Blue staining in model rats demonstrated severer distortion of the lung structure and larger fibrous area compared to normal rats and rats administrated with sunitinib; (**C**–**G**) showed an serious larger bronchiole tube area (**C**), thicker tube wall (**D**), increased MA% (E), MT% (**F**) and higher level of histological score (**G**) in COPD model compared to the normal rats (**P < 0.01, *P < 0.05, compared to the normal rats), which could be effectively improved by sunitinib (^##^*P* < 0.01, ^#^*P* < 0.05, compared to the untreated COPD rats).

Histological examination of COPD lungs revealed the presence of disordered lung architecture and pulmonary fibrosis (Fig. 1A,B). LPS injection and chronic CS results in structural damage of lung with septal congestion, epithelial thickening and parenchyma proliferation, numerous inflammatory cells, neutrophils, macrophages, and lymphocytes accumulated in both the alveolar walls and spaces. Serious larger bronchiole tube area, thicker tube wall surrounded by proliferative connective tissue, and increased MA%. MT% were obviously detected compared to the normal rats (**P = 0.000 < 0.01) (Fig. 1C,F). Additionally, sunitinib untreated COPD rats showed a 3-fold increase in the injury score (Fig. 1G) for most of them exhibited septal congestion, epithelial thickening, septal inflammatory infiltrates, alveolar haemorrhage or massive disruption of lung structure, while normal rats showed integrated alveolar walls and no obvious infiltration of inflammatory cells or histological damage to small, and sunitinib administrated rats exhibited changes somewhere in between, with a slightly fracture of alveolar wall and mild infiltration of inflammatory cells, surprisingly, there were less changes in tube wall of bronchiole.

### The phenomenon of angiogenesis in COPD rats

**Figure 2:**
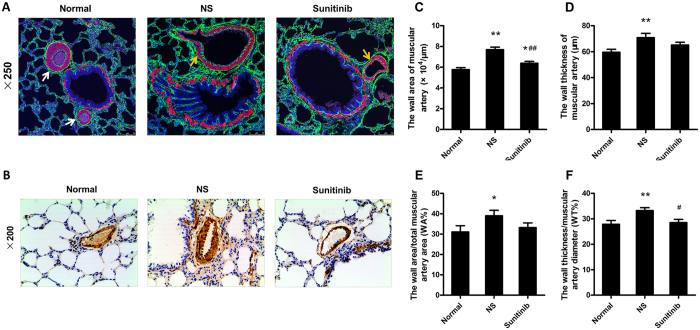
Vasculature changes in COPD rats. (**A**) Histology of vasculature related cells and vascular density. Vascular endothelial cell was stained with immunofluorescence technique, CD31 (green), vascular smooth muscle cell and fibroblast, myfibroblast, α-SMA (red) and nucleus staining (blue) (original magnification ×250). (It is demonstrated that angiogenesis happened in COPD rats and can be effectively attenuated by sunitinib administrating. Here white arrowheads point to small arteries and the yellow one points to the small veins. (**B**) showed strong immunolabelling of α-SMA (brown) expressed in the muscular arteriesand abundant α-SMA (brown) presented in the accompanied bronchiole and sunitinib decreased the expression level (immunohistochemistry staining; original magnification ×200). (**C**–**F**) showed the COPD rats had the larger tube wall area of muscular artery (**C**), the thicker tube wall thickness (**D**) and higher WA% (**E**), WT% (**F**) compared to the normal rats (**P < 0.01, *P < 0.05, compared to the normal rats), while sunitinb intervention resulted in lesser tube wall area of muscular artery (**C**) and lower WT% (**F**) compared to rats disposed on NS, but tube wall thickness (**D**) and WA% (**E**) of muscular artery showed no difference (^##^*P* < 0.01, ^#^*P* < 0.05, compared to the untreated COPD rats).

Angiogenesis is also a protruding feature of COPD rats. We localized CD31 and α-SMA expression in lung tissue. CD31 is the marker of vascular endothelial cell and α-SMA can represents both vascular smooth muscle cell and fibroblast, myfibroblast cell. Figure 2 A provides a selected representative images of lung sections in each group. The CD31 and α-SMA positive cells, showed in green and red respectively, both increased in bronchiole of sunitinib untreated COPD rats compared to the normal rats and sunitinib administrated rats (Fig. 2A). In addition, strong immunolabelling of α-SMA expressed in muscular arteries that companied with bronchiole (Fig. 2B) and sunitinib had a decreasing effect on its expression level. Quantification by image analysis software (image-proplus, Version 5.0) showed that the COPD rats had the larger tube wall area of muscular artery (Fig. 2C, *P* = 0.000 < 0.01), the thicker tube wall thickness (Fig. 2D, *P* = 0.005 < 0.01), higher WA% (Fig. 2E, *P* = 0.000 < 0.01), and WT% (Fig. 2F, *P* = 0.000 < 0.01) compared to the normal rats, while sunitinb intervention resulted in lesser tube wall area of muscular artery (Fig. 2C, *P* = 0.000 < 0.01) and lower WT% (Fig. 2F, *P* = 0.017 < 0.05) compared to untreated rats, but tube wall thickness and WA% of muscular artery showed no difference.

### VEGF, one of the most important factors in promoting angiogenesis, high expression level lead to severer small-airway remodelling

**Figure 3:**
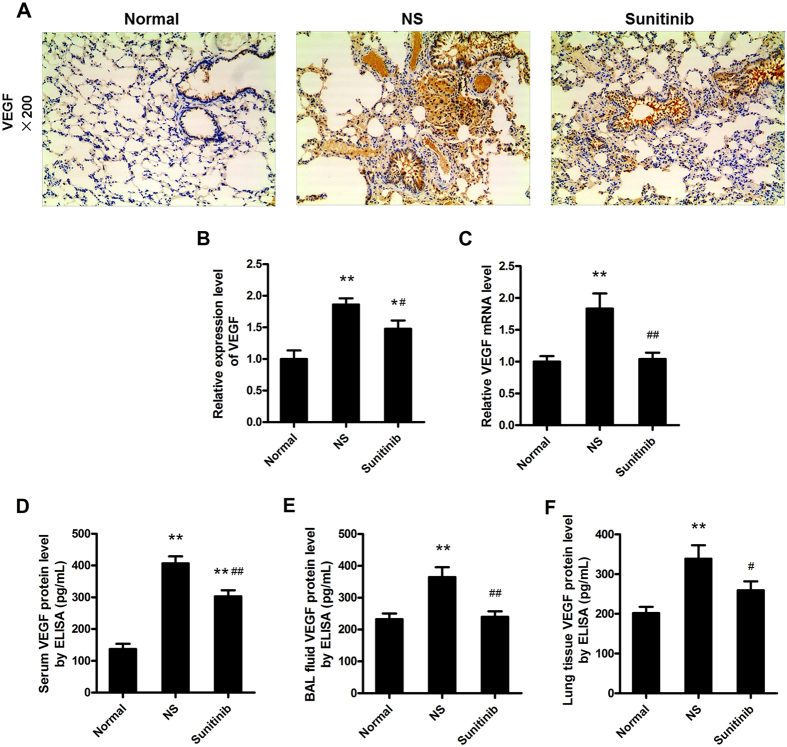
VEGF expression in COPD rats. (**A**) showed the strongest immunolabelling of VEGF (brown), that was present in the tracheal epithelium, vascular smooth muscle cells, infiltrated inflammatory cells, and phagocytic cells (immunohistochemistry staining; original magnification ×200). (**B**) showed the relative expression level of VEGF of COPD groups were obviously higher than normal rats and decresed in rats dealt with sunitinib. (**C**) untreated COPD rats can express higher VEGF mRNA level in lung tissue homogenates, which can be significantly inhibited by sunitinib. (**D**–**F**) demonstrated higher level of VEGF in the serum, BAL fluid and lung tissue that related to the small-airway remodelling, and can be inhibited with sunitinib (**P < 0.01, *P < 0.05, compared to the normal rats), (^##^*P* < 0.01, ^#^*P* < 0.05, compared to the untreated COPD rats).

Rats administrated with NS showed the strongest immunolabelling of VEGF, that was present in the tracheal epithelium, vascular smooth muscle cells, infiltrated inflammatory cells, and macrophage cells (Fig. 3A). Compared to this group, VEGF immunolabelling in rats dealt with sunitinib, was obviously decresed (Fig. 3B, *P* = 0.017 < 0.05). Figure 3C provided the evidence that COPD rats can express higher VEGF mRNA level in lung tissue (Fig. 3C, *P* = 0.000 < 0.01), which can be significantly inhibited by sunitinib (Fig. 3C, *P* = 0.001 < 0.01).

ELISA result showed that the serum concentrations of VEGF were considerably higher in the untreated COPD rats than normal rats (Fig. 3D, *P = 0.000* < 0.01), while rats treated with sunitinib maintained moderate levels. The levels of VEGF were also considerably different in BAL fluid and lung tissue. The untreated model rats had significant higher VEGF level in BAL fluid and lung tissue than those in normal group (Fig. 3E, *P = 0.000* < 0.01) (Fig. 3F, *P = 0.001* < 0.01), respectively. However, it is detected that the rats disposed on sunitinib had the lower expression level of VEGF level in both BAL fluid and lung tissues compared to the untreated model rats (Fig. 3E, *P = 0.001* < 0.01) (Fig. 3F, *P = 0.033* < 0.05), which stayed the same expression level as the normal rats.

### VEGF exerts effect via specific binding to cell surface expressed receptors, VEGFR1 and VEGFR2, which can be partly inhibited by sunitinib

**Figure 4:**
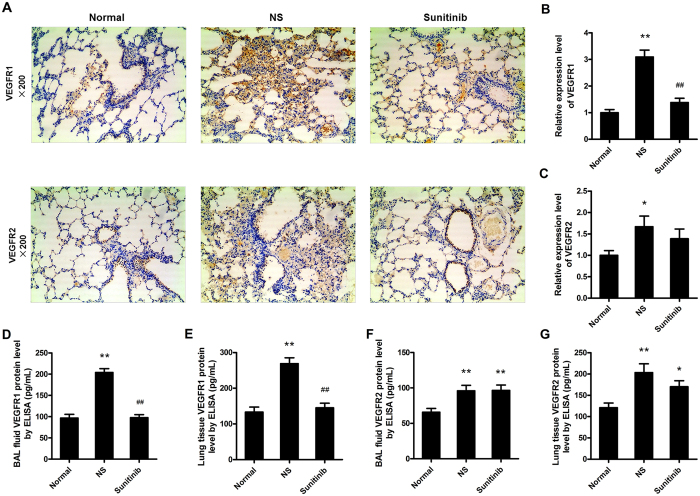
VEGFR1 and VEGFR2 roles and changes in airway remodelling. (**A**) provided the data that moderately strong immunolabelling of VEGFR1 (brown) expressed in the epithelial cells of the respiratory vessels and a relatively lower level of VEGFR2 (brown) was discovered in the bronchial epithelial cells, capillary endothelial cells, and a small number of vascular smooth muscle cells (immunohistochemistry staining; original magnification ×200). (**B**) indicated a relative higher level of VEGFR1 in COPD rats compared the to normal rats and can be partly reversed with sunitinib. (**C**) showed a relative higher level of VEGFR2 in COPD rats, but sunitinib couldn’t inhibit it. (**D**,**E**) the concentration of VEGFR1 increased in BAL fluid and lung tissue in COPD rats and can be inhibited with sunitinib. (**F**,**G**) the level of VEGFR2 gained in the BAL fluid and lung tissue of both COPD groups (**P < 0.01, *P < 0.05, compared to the normal rats), (^##^*P* < 0.01, ^#^*P* < 0.05, compared to the untreated COPD rats).

Moderately strong immunolabelling of VEGFR1 was present in the epithelial cells of the respiratory vessels. A relatively lower level of VEGFR2 was discovered in the bronchial epithelial cells, capillary endothelial cells, and a small number of vascular smooth muscle cells (Fig. 4A). In addition, quantification in immunohistochemical analysis, the untreated COPD group showed a higher expression level of VEGFR1 than did the normal and sunitinib administrated groups (Fig. 4B, *P* = 0.000 < 0.01), in which the sunitinib treated group stayed the same level as the normal group. Sequencely, the relatievely lower level of VEGFR2 can be tested in normal rats, compared to untreated COPD rats (Fig. 4C, *P* = 0.030 < 0.05), and it suggested no difference between rats dealt with NS or sunitinib.

The BAL fluid concentrations of VEGFR1 were considerably higher in the COPD rats treated with NS than normal rats (Fig. 4D, *P* = 0.000 < 0.01), while rats treated with sunitinib maintained moderate levels. The levels of VEGFR1 were also considerably different in lung tissue. The NS disposed rats had highest VEGFR1 levels among three groups), and sunitinib administrated rats ranked in between (Fig. 4E, *P* = 0.000 < 0.01). In contrast, the VEGFR2 concentrations in BAL fluid and lung tissue were similar between the two COPD groups, although they were expressed higher levels than normal rats (Fig. 4F,G).

## Discussion

COPD is a leading cause of chronic morbidity and mortality worldwide, that is characterized by mucus hypersecretion, small-airway remodelling, epithelial cell hypertrophy, emphysematous changes and other events. LPS intratracheal injection and chronic CS inhalation models of COPD also exhibit these feature and have been developed successfully in rats models^7^,^8^. Chronic small-airway remodelling, features with thickened tube wall and connective tissue proliferation, is thought to be the most essential pathological feature in the disease progression^9^. In the last century, extensive studies have shown that chronic inflammation is the most correlative factor attributed to airway remodelling; in more recent decades, a mechanism of small-airway remodelling based on angiogenesis has been given increasingly more attention by experts worldwide^10^,^11^.

Angiogenesis, first discovered by Leonardo da Vinci^12^, refers to new capillary growth caused by proliferation and migration of vascular endothelial cells on the basis of the original capillary and/or the micro vein. This is a vigorous and continuous process^13^ that may occur in various organs and tissues, always related to the development of various cancer^14^,^15^ and metabolic disease, such as insulin resistance, obesity^16^. The vasculature in respiratory system plays an essential role in maintenance of physiological functions in lung for the great plasticity and expansive capacity, and a variety of pathological changes may occur if frequent or long-term harmful smoke, industrial poison exposure, or chronic inflammation, including increased vascular endothelial cell permeability, more expression of adhesion molecules, proliferation and migration of capillary endothelial cells, and infiltration of inflammatory cells, which could result in the formation of new blood vessels^17^.

In the present study, the rat model of COPD was established by LPS intratracheal injection and chronic CS inhalation, HE and Masson staining were assessed to evaluate pulomonary pathological changes, especially the small-airway structure and final remodelling. COPD rats had larger tube wall area and higher tube wall thickness of both the bronchioles and muscular arteries than normal rats, as well as a totally destroyed lung structure surrounded by proliferative connective tissue and demolished alveoli with marked inflammatory cell infiltration. Our results also demonstrate that, in the present experimental models, angiogenesis and vascular remodelling can be rapidly achieved and confirmed by the immunostaining techniques.

During the process of angiogenesis, various angiogenic growth factors including VEGF, fibroblast growth factor^18^, angiogenin^19^, placenta growth factor^20^, and tie2 and its ligands angiopoietin-1 and angiopoietin-2^21^ are involved in effective and durable regeneration and reconstruction of blood vessels. Among these growth factors, VEGF is thought to be the most important factors in angiogenesis^22^ especially in the chronic inflammatory process, such as asthma^23^.

Studies also showed VEGF exerts biological effects^24^ via specific binding to cell surface expressed receptors, VEGFR-l (flt-1), or VEGFR2 (flk-1/KDR), which equipped with tyrosine kinase activity^25^. The activation of receptor kinase activity allows coupling to downstream signal transduction pathways that participates in the critical rate-limiting step of physiological angiogenesis by increasing the permeability of the microvasculature, promoting the proliferation, migration and differentiation of endothelial cells. VEGFR1, restricted to endothelial cells, can release tissue-specific growth factors possibly in a vascular bed-specific fashion, and VEGFR2 is a major mitogenic and angiogenic mediator that can enhance the permeability-inducing effects of VEGF^26^. Thus, the levels of VEGF, VEGFR1, VEGFR2 are the credible indicators to evaluate the degree of angiogenesis. In our study, the elevated lever of VEGF and VEGFR1, VEGFR2 presented ongoing evidence of increased angiogenic activity and proved that VEGF activity related angiogenesis and microvascular remodelling in the airways are the parts of structural airway remodelling in the COPD model, which is similar to the clinical research about asthma and emphysema patients^27^,^28^.

However, we must point out the weakness of VEGFR2 as an indicator of angiogenesis progression because of its minimal variation in the BAL fluid and lung tissue between the two COPD groups that measured by the ELISA kits, as well as the lower expression level of VEGFR2 in lung tissue that tested by immunohistochemical analysis. Similar to the Feltis BN^28^ and Wang K^29^’s researches, we tend to support the idea that VEGFR1 played more crucial role in angiogenesis through combining with VEGF.

The another significant finding of the present study is the efficacy of sunitinib for inhibition of angiogenesis in small-airway remodelling in rats of COPD. Sunitinib is a multi-targeted protein-tyrosine kinase inhibitor that have been approved by the Food and Drug Administration for the first-line treatment options of clear-cell, metastatic renal-cell carcinoma^30^. It is reported that sunitinib can inhibit at least eight receptor protein-tyrosine kinases including VEGFR1-VEGFR3, platelet-derived growth factor receptors (PDGFRa and PDGFRb), stem cell factor receptor (Kit), Flt-3, and colony-stimulating factor-1 receptor (CSF-1R). Sunitinib effectively interfere with the process of tumour angiogenesis by primarily diminishing signaling through VEGFR1, VEGFR2, which may indirectly reducing the level of VEGF, VEGFR1 and VEGFR2^31^. In our sunitinib treatment study, we noted after 1 month administration, the lung architecture was better than that of rats treated with NS, with mild infiltration of inflammatory cells, only slightly proliferated connective tissue and disordered architecture with less thicker bronchioles and muscular arteries tube wall. The intensity of immunolabeling for VEGF, VEGFR1, VEGFR2 and α-SMA were also clearly lower in the sunitinib administrated rats. Moreover, the levels of VEGF in the serum and the levels of VEGF, VEGFR1 in BAL fluid and lung tissue of the sunitinib-treated rats all were lower than those in untreated rats. Finally, we can get an identical consequence that the COPD small-airway remodelling related to the angiogenesis promoted by higher expression level of VEGF, VEGFR1, VEGFR2. Similarly, the impact of sunitinib on VEGFR2 expression level in COPD rats seemed inconspicuous, which was also a compelling evidence to support the idea that VEGFR2.

In conclusion, the results of the present study demonstrate that VEGF plays a vital important role in the process of small-airway remodelling in a rat model of COPD through facilitating angiogenesis, which mainly depend on the specific binding between VEGF and VEGFR1 and can be effectively attenuated by the treatment of sunitinib.

However, our study had few limitation, more technology, such as immunoblotting, genetic manipulation method, and phosphorylation level of VEGFR1 and VEGFR2 may be done to support our conclusion. Also, more standardized and rigorous studies on other angiogenesis related factorsin small-airway remodelling may be further performed.

## Methods

### Ethical approval

#### Approval

The experimental protocol was approved by the Animal Care Committee of Zhejiang Chinese Medical University (Hangzhou, China).

#### Accordance

The methods were carried out in accordance with the approved guidelines.

### Animals preparation

A total of 39 male Sprague-Dawley rats (180 to 220 g) were included. All rats were housed five per cage in an animal care room of Animal Experimental Center of Zhejiang Chinese Medical University, with a temperature and relative humidity maintained within the target range of 18 °C ± 22 °C and 40% to 70%, respectively, and a 12-h light/dark cycle. Food and water were fed ad libitum except when rats were placed in the self-made cigarette smoke box.

### Experimental design and sunitinib administration

Animals were initially randomized to two groups: non-COPD controls (n = 13) and COPD (n = 26). COPD was induced by LPS injection and CS inhalation. LPS is considered the gold standard model for acute pulmonary inflammation^32^ and CS is thought to be the most critical risk factor in the development of COPD. A detailed description of this model is that animals were challenged with a single intratracheal dose of 0.2 mg/ml/kg LPS (Escherichia coli O55:B5, L2280, Sigma, Louis, MO, USA) on days 30 and 45, and with CS inhalation^33^ in a self-made cigarette smoke box every 12 hours on all days except days 30 and 45. Thirty cigarettes were used each time to ensure a smoke concentration of 450 to 500 bpm. At week 20, three rats were chosen from each groups randomly and HE staining was performed to evaluate the model that whether it meet the real histological conditions in patients with COPD. COPD animals received intragastric administration of sunitinib (H0125803, Roche, Reinach, Switzerland), or 0.9% normal saline, at a dosage of 10 mg/kg once daily, respectively^34^,^35^. Non-COPD group was not accepted CS inhalation or medication. All rats were fed a normal diet during the drug administration period and were killed after 1 month.

### Blood sample collection

Animals were anesthetized by intraperitoneal injection of ketamine (50 mg/kg) and xylazine (2 mg/kg). The peritoneal cavity was opened and the abdominal aorta was gently separated form the surrounding connective tissue and fat. Blood samples (5 ml) were obtained from the abdominal aorta by indwelling needle and were centrifuged for 15 min at 1500 g. The aliquots were collected into Eppendorf tubes and preserved at -80 °C for subsequent ELISA.

### Preparation of bronchoalveolar lavage fluids

A cervical tracheotomy was performed using a 14 G catheter. The BAL fluid was obtained by cannulating the trachea and lavaging with 1 ml sterile 0.9% saline on a restraining board inclined 45° from the horizontal. Roughly 0.7- 0.8 ml of BAL fluid could be recovered and repeated three times to get a total of 2.1 to 2.3 ml of BAL fluid. The recovered BAL fluid was centrifuged for 5 min at 1000 g to separate the cell precipitation and cell-free fluid. The cell-free lavage fluid was stored at −80 °C used for subsequent ELISA.

### Histological study

The left lung was isolated, fixed intratracheally with 2 ml 4% formaldehyde, washed with PBS, and immersed in the same fixative for at least 24 h. After formaldehyde-fixed, paraffin-embedded, the specimens sectioned at 3–4 μm and processed for a standard hematoxylin-eosin staining and Masson-Goldner trichrome technique. Slides were viewed and photographed with the fluorescence microscope (BX20, Olympus, Tokyo, Japan) at ×100 and 200 magnification, respectively. A semi-quantitative morphometric analysis of lung injury was determined by particular histological score, according to the following scale: 0, normal lung; 1, septal congestion; 2, epithelial thickening; 3, septal inflammatory infiltrates; 4, alveolar hemorrhage and/or hyaline membranes; 5, massive disruption of lung architecture^36^. Additionally, three random microscope fields in which the bronchiole diameters were <100 μm (shortest path/lumen diameter, ≥0.7) at a magnification of ×100 were observed to determine the changes of thickness and area of the tube wall. The wall area/total bronchiole area (MA%) and the wall thickness/bronchiole diameter (MT%) were then calculated. Also, a muscular artery diameter of 50 to 100 μm was selected to evaluate small vessel remodelling at a magnification of ×200. The wall area/total muscular artery area (WA%) and the wall thickness/muscular artery diameter (WT%) were also computed.

### Immunostaining studies

The sections of left lung were also used for immunohistochemistry analysis. Fully processed with 0.01 m citrate buffer (pH 6.9) and 3% H_2_O_2_, the random sections were stained antibodies against VEGF (1:400, 2142671, Millipore, Billerica, USA), VEGFR1 (1:200, Ab32152, Abcam, Cambridge, USA), VEGFR2 (1:75, Ab131441, Abcam, Cambridge, USA) andα-SMA (1:250, M0851, 1A4, Dako, Glostrup, Denmark), respectively. The number of VEGF, VEGFR1, VEGFR2 positive cells was counted in three randomly chosen ×100 fields by semi-quantitative method that number of positive number multiplied by positive intensity. The fresh tissues of anterior and middle lobes of right lung were paraformaldehyde-fixed and paraffin-embedded in the same way, and proceeded for whole-mount immunofluorescence. Using a standard protocol, the samples stained at 37 °C for 1 hour with a mixture of rabbit anti-mouse CD31 antibody (1:50, Ab28364, Abcam, Cambridge, USA) and rat anti-mouse α-SMA antibody (1:100, 1A4, Dako, Glostrup, Denmark). Subsequently, tissue samples were thoroughly washed and vascular endothelial cells were detected with the mixture of a secondary goat anti-rabbit FITC-labeled antibody (1:60) and secondary goat anti-rat TRITC-labeled antibody (1:60). After rigorous incubating and washing, tissue samples were stored in the dark at −20 °C until positive signals were observed using a confocal microscope (Leica, TCS SP8 STED, Germany).

### Enzyme-linked immunosorbent assay (ELISA)

To investigate the effect of VEGF, VEGFR1, VEGFR2 in small-airway remodelling, the VEGF concentrations in the serum, BAL fluid, lung tissue homogenates, and the contents of VEGFR1, VEGFR2 in the BAL fluid and lung tissue, either normal groups, or rats dealt with sunitinib, all were measured using a sensitive VEGF ELISA kit (EIA06628r, Revelation Biotechnology Co. Ltd, Wuhan, China), VEGFR1 ELISA kit (EIA06629r, Revelation Biotechnology Co. Ltd, Wuhan, China), and VEGFR2 ELISA kit (EIA06630r, Revelation Biotechnology Co. Ltd, Wuhan, China) according to the instructions of the manufacturer, respectively. VEGF, VEGFR1, VEGFR2 concentrations were expressed as pg/ml.

### RNA extraction and quantitative real-time PCR

TRIzol^®^ Plus RNA Purification Kit (12183-555, Invitrogen, Carlsbad, USA) was used for total RNA extraction from the remaining accessory lobe of lung tissue and 40–100 ul RNase-Free DNase Set (79254, Qiagen, GmBH, German) was served to avoid extracted RNA degradation and remove genomic DNA contamination. First-strand cDNA was synthesized with the SuperScript™ III First-Strand Synthesis SuperMix (11752-050, Invitrogen, Carlsbad, USA) according to the manufacturer’s instructions, 100 ng-1 μg aliquots of the total extracted RNA was treated with 10 μl 2 × RT Reaction Mix, 2 μl RT Enzyme Mix and certain RNase-Free Water to a total volume of 20 μl. The PCR amplification was performed by using 10 μl Power SYBR^®^ Green PCR Master Mix (4367659, Applied Biosystems, Austin, USA), 1 μl synthesized cDNA, 8 μl RNase-Free Water, and 0.5 μl of Forward Primer and Reverse Primer, respectively, to a total volume of 20 μl. All PCR reactions were run in a Multiplex Real Time PCR machine (CFX384, Bio-Rad, CA, USA). After an initial denaturation step at 95 °C for 1 min, the protocol was executed for 40 cycles as follows: denaturation at 95 °C for 15 sec, annealing at 63 °C for 25 sec, and extension at 72 °C for 45 sec. The primer sequence was desgined by Primer Premier 6.0 and Beacon designer 7.8, and specific for VEGF genes is 5′- GTCACCACCACACCACCATCGT -3′ and 5′-CTCCTCTCCCTTCATGTCAGGCT-3, 76 bp; Rat 18 s primer sequence is 5′-GAATTCCCAGTAAGTGCGGGTCATA-3′, 5′- CGAGGGCCTCACTAAACCATC-3′, 105 bp. The amplification program is followed by melting curve analysis. A negative control was included in each run to evaluate the specificity of primers and possible contamination. The relative quantification of VEGF mRNA was calculated using the 2^−ΔΔCt^ method^37^.

### Statistical Analysis

All data are presented as mean ± standard error. The normality of distribution was evaluated with the Kolmogorov-Smirnov test. Comparisons between groups were performed with one-way ANOVA for normally distributed data and with the Kruskal-Wallis tests for skewed data. Values of *P* < 0.05 were considered statistically significant. Statistical analysis was performed using SPSS 19.0 (IBM Corp, Armonk, NY, USA).

## Additional Information

**How to cite this article**: Wang, L. *et al*. The Role of Vascular Endothelial Growth Factor in Small-airway Remodelling in a Rat Model of Chronic Obstructive Pulmonary Disease. *Sci. Rep.* **7**, 41202; doi: 10.1038/srep41202 (2017).

**Publisher's note:** Springer Nature remains neutral with regard to jurisdictional claims in published maps and institutional affiliations.
